# Targeted delivery of fluorogenic peptide aptamers into live microalgae by femtosecond laser photoporation at single-cell resolution

**DOI:** 10.1038/s41598-018-26565-4

**Published:** 2018-05-29

**Authors:** Takanori Maeno, Takanori Uzawa, Izumi Kono, Kazunori Okano, Takanori Iino, Keisuke Fukita, Yuki Oshikawa, Taro Ogawa, Osamu Iwata, Takuro Ito, Kengo Suzuki, Keisuke Goda, Yoichiroh Hosokawa

**Affiliations:** 1 Graduate School of Materials Science, Nara Institute of Science and Technology, Ikoma, 630-0192 Japan; 2 Nano Medical Engineering Laboratory, RIKEN, Wako, 351-0198 Japan; 3 RIKEN Center for Emergent Matter Science, Wako, 351-1098 Japan; 4 euglena Co., Ltd, Yokohama, 230-0046 Japan; 5 Japan Science and Technology Agency, Kawaguchi, 332-0012 Japan; 6 Department of Chemistry, University of Tokyo, Tokyo, 113-0033 Japan

## Abstract

Microalgae-based metabolic engineering has been proven effective for producing valuable substances such as food supplements, pharmaceutical drugs, biodegradable plastics, and biofuels in the past decade. The ability to accurately visualize and quantify intracellular metabolites in live microalgae is essential for efficient metabolic engineering, but remains a major challenge due to the lack of characterization methods. Here we demonstrate it by synthesizing fluorogenic peptide aptamers with specific binding affinity to a target metabolite and delivering them into live microalgae by femtosecond laser photoporation at single-cell resolution. As a proof-of-principle demonstration of our method, we use it to characterize *Euglena gracilis*, a photosynthetic unicellular motile microalgal species, which is capable of producing paramylon (a carbohydrate granule similar to starch). Specifically, we synthesize a peptide aptamer containing a paramylon-binding fluorescent probe, 7-nitrobenzofurazan, and introduce it into *E. gracilis* cells one-by-one by suppressing their mobility with mannitol and transiently perforating them with femtosecond laser pulses at 800 nm for photoporation. To demonstrate the method’s practical utility in metabolic engineering, we perform spatially and temporally resolved fluorescence microscopy of single live photoporated *E. gracilis* cells under different culture conditions. Our method holds great promise for highly efficient microalgae-based metabolic engineering.

## Introduction

By virtue of the excellent phylogenetic and phenotypic diversity of microalgae, microalgae-based metabolic engineering has been proven effective for producing valuable substances for food, medicine, and energy in the past decade^1–5^. It involves the use of cultivation techniques and genetic engineering to optimize the metabolism of microalgae by altering their biochemical pathways and introducing new pathway components into live microalgae^1,2,6,7^. Popular microalgal species for metabolic engineering are *Botryococcus braunii*, *Chlamydomonas reinhardtii*, *Chlorella* sp., *Dunaliella tertiolecta*, *Euglena gracilis*, *Nannochloropsis* sp., and *Spirulina* as economically viable sources of bioactive compounds such as fatty acids, carotenoids, sterols, phycobilins, pectins, toxins, and halogenated compounds^1–3,6–9^. The products that have been produced by these microalgal species to date include food supplements^3^, pharmaceutical drugs^3,4,10^, biodegradable plastics^7^, and biofuels^5,7^. Microalgae offer a few clear advantages in metabolic engineering over plants and other microorganisms. First, microalgae require minimal environmental resources because a majority of them can grow both autotrophically and heterotrophically^7,11^. Second, they have evolved to tolerate and adapt to a broad range of environments including saline and wastewater anywhere between the equator and poles^12^. Finally, they are expected to be a potential solution to global warming since they are known to consume about 40% of CO_2_ on Earth via photosynthesis and produce neutral oil that can be used as a biofuel alternative to fossil fuels^12^.

For efficient metabolic engineering, it is critical to accurately visualize and quantify intracellular metabolites within single live microalgal cells, but remains a major challenge due to the lack of proper characterization methods^13^. Population-averaged ensemble measurements based on high-performance liquid chromatography^14,15^, electrophoresis^16^, mass spectrometry^17^, and microplate readers^18^ are useful for identifying and quantifying chemical substances in a mixture, but fail to probe cellular heterogeneity which is normally amplified by genetic engineering and environmental perturbations. Conjugated antibodies linked to fluorescent dyes (e.g., BODIPY and Nile Red) for fluorescence microscopy^19^ are effective for single-cell analysis of live microalgae, but are too large to penetrate through their cell wall and probe intracellular metabolites. While Raman microscopy^20^ has been recently shown useful for imaging intracellular metabolites at single-cell resolution, its molecular specificity is relatively low since it cannot differentiate similar types of molecules with similar vibrational signatures such as proteins and polysaccharides. Therefore, there is an immediate need for overcoming these problems and characterizing metabolites in live microalgal cells at single-cell resolution for efficient metabolic engineering.

In this Article, we demonstrated the synthesis of fluorogenic peptide aptamers with specific binding affinity to a target metabolite and the targeted delivery of them into live microalgae by femtosecond laser photoporation^21–25^ for the accurate visualization and quantification of their intracellular metabolites at single-cell resolution. Since aptamers are small combinatorial polypeptides (much smaller than antibodies) which are typically composed of a variable peptide region of 8–20 amino acids in length, they can be effectively delivered into live microalgae through their cell wall by photoporation. Furthermore, their fluorogenic property and specific binding affinity allowed us to visualize the spatial and temporal accumulation of metabolites in live microalgae. As a proof-of-demonstration of our characterization method, we used it to characterize *Euglena gracilis*, a photosynthetic unicellular motile microalgal species, which has been studied as a model organism for decades and is considered commercially attractive due to its mass producibility and ability to produce paramylon (β-1,3-glucan or a carbohydrate similar to starch)^26^. Paramylon is a unique substance produced only by Euglenids including the genus *Euglena* with functions similar to those of roughage or dietary fiber and is converted within the cell into wax esters which can be refined to produce kerosene suitable for biofuels^27^. To characterize the production of intracellular paramylon in *E. gracilis* cells spatially and temporally, we synthesized a peptide aptamer containing a fluorescent probe, 7-nitrobenzofurazan (NBD), which emits fluorescence upon binding to paramylon, and introduced it into *E. gracilis* cells in culture medium one-by-one by suppressing their mobility with mannitol added to the culture medium and transiently perforating them with amplified femtosecond laser pulses at 800 nm for photoporation^22,24^. To verify the efficient delivery and binding of the aptamer to paramylon at single-cell resolution, we performed fluorescence microscopy of the single photoporated cells with patterned femtosecond laser excitation. Specifically, we monitored the time-varying distribution of intracellular paramylon in the cells under glucose-sufficient and -deficient conditions.

## Results

### Paramylon-binding fluorogenic peptide aptamers

**Figure 1:**
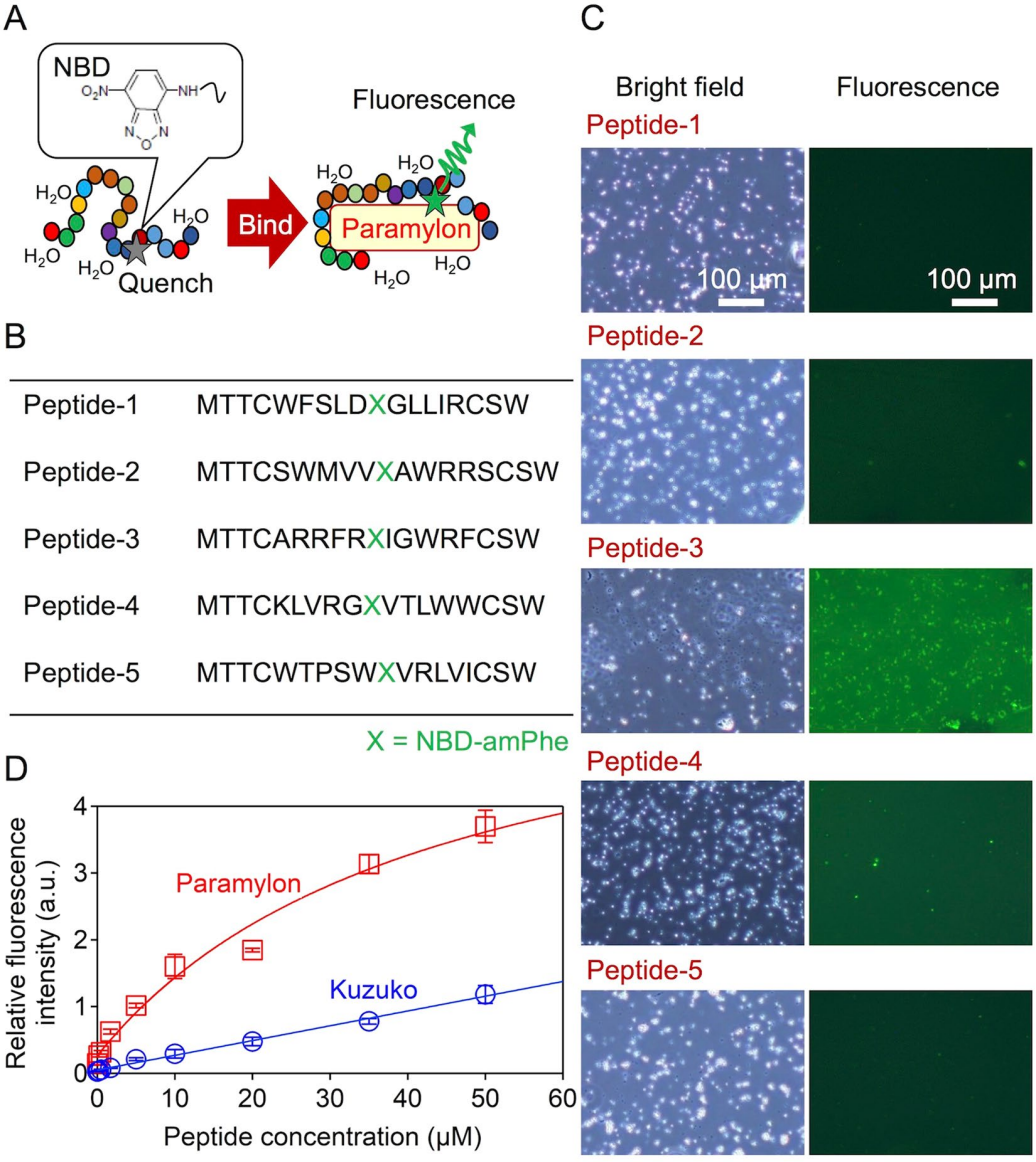
Paramylon-binding fluorogenic peptide aptamer. (**A**) Principle of the binding and fluorescence of the aptamer. (**B**) Peptide sequences selected by ribosome display. (**C**) Bright-field and fluorescence images of paramylon granules to which the peptide aptamer was bound. Although we used the same settings to obtain these images, pepide-3 exhibited a higher background signal than the other peptides, presumably because the micro-environment of the NBD in peptide-3 was relatively hydrophobic. (**D**) Relation between the fluorescence intensity from paramylon and Kuzuko and the concentration of peptide-3, which we refer to as the fluorogenic paramylon-binding peptide (FPBP). The red and blue curves are fits to the data points with the Langmuir equation. The nonlinear relation is due to the occupation of the binding sites on paramylon granules by the FPBPs, indicating that the FPBPs specifically bind to paramylon granules.

To generate a fluorogenic paramylon-binding peptide, we employed ribosome display^28^ with modification to incorporate a fluorogenic amino acid^29^ (Supplementary Fig. 1). We redefined the TAG stop codon to a sense codon by eliminating release factor 1 from the *in vitro* translation system^30^ and alternatively adding the tRNA_CUA_ which was coupled with aminophenylalanine (amPhe) modified with NBD. We employed NBD as a source of the fluorogenic property of a peptide aptamer because the florescence intensity of the NBD is environment-sensitive^31^, such that the fluorescence of the NBD is effectively quenched in a hydrophilic environment whereas the fluorescence is emitted in a hydrophobic environment (Fig. 1A). After seven rounds of ribosome display, we found seven sequences more than twice in 51 cloned sequences (Supplementary Table 1). While two sequences among the seven sequences met failure of synthesis presumably due to their poor solubility, we synthesized five peptides by solid phase chemistry (Fig. 1B).

Next, we examined the fluorogenic property and specificity of these five peptides against paramylon granules. As shown in Fig. 1C, the peptide-3 peptide, which we refer to as the fluorogenic paramylon-binding peptide (FPBP), emits fluorescence upon binding to paramylon granules whereas the other four peptides do not, which clearly indicates that only the FPBP possesses the fluorogenic property against paramylon granules. Figure 1D shows the comparison in binding specificity to paramylon between the FPBP and starch (Kuzuko, a starch powder extracted from the root of the kudzu plant, *Pueraria lobata*) which is the most widespread and abundant metabolite in microalgae and plants^32^, but is not produced in *E. gracilis*^33,34^. While starch consists of glucose similar to paramylon, the FPBP emits effectively stronger fluorescence when binding to paramylon granules than starch (Fig. 1D). This difference presumably arises from structural differences in these two polysaccharides that starch is α-1,4-glucan with α-1,6 branches while paramylon is β-1,3-glucan. The specificity of the FPBP was further verified by measuring no increase in the fluorescence signal level as the concentration of the monomer unit of these polysaccharides, glucose, and triglucose (maltotriose) was changed (Supplementary Fig. 2).

### Procedure for targeted delivery of fluorogenic peptide aptamers into live *Euglena gracilis* cells

**Figure 2:**
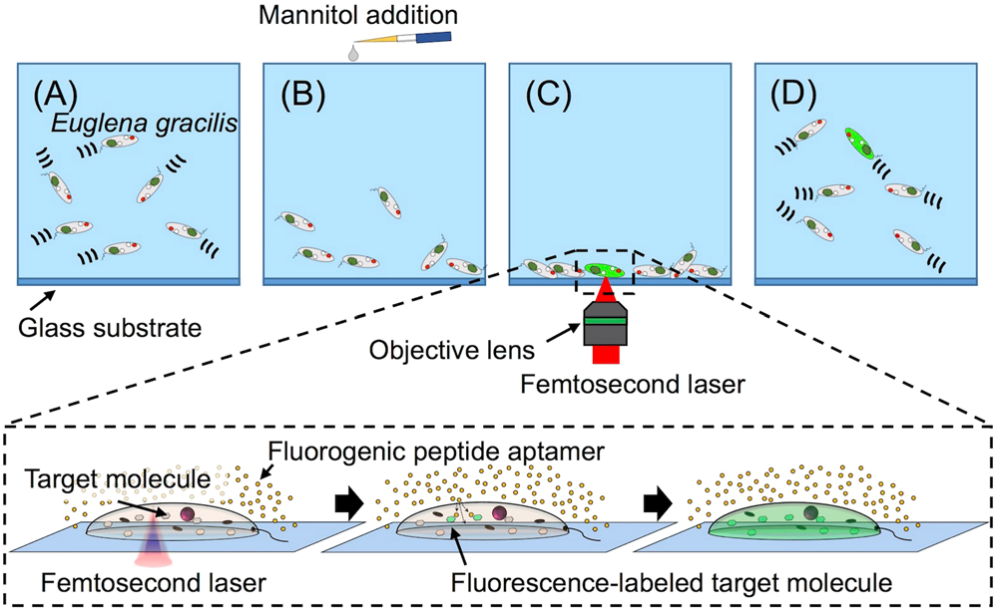
Procedure for the targeted delivery of the FPBP into live *E. gracilis* cells. In Step (**A**) *E. gracilis* cells are cultivated in culture medium. In Step (**B**) mannitol is added to the culture medium to increase osmotic pressure on the cells and suppress their mobility. In Step (**C**) the cells are transiently perforated one-by-one by femtosecond laser photoporation to inject the aptamer into the cells. In Step (**D**) the cells restore their mobility and freely move a few hours after the photoporation.

We performed the targeted delivery of the FPBP into *E. gracilis* cells with the following procedure (Fig. 2). In the first step (Step A in Fig. 2), we cultivated *E. gracilis* cells on a petri dish for at least 6 days under 14/10 light/dark cycle illumination at 26 °C. Active cells were able to freely swim with their flagellates in the medium. In the second step (Step B in Fig. 2), we added the FPBP (50 µM) and mannitol (0.3 M) to the medium in order to increase osmotic pressure on the cells for photoporation and suppress their mobility so that the cells sank to the bottom of the petri dish. In the third step (Step C in Fig. 2), we focused femtosecond laser pulses (800 nm, 100 fs) onto each cell through a 20× objective lens (NA. 0.45) under an inverted microscope to transiently perforate the cells. The FPBP was injected through the produced small holes into the cells by the osmotic pressure. In the final step (Step D in Fig. 2), the cells restored their mobility and were able to freely swim a few hours after the addition of mannitol into the medium.

### Suppression of the mobility of *Euglena gracilis* cells by mannitol addition

**Figure 3:**
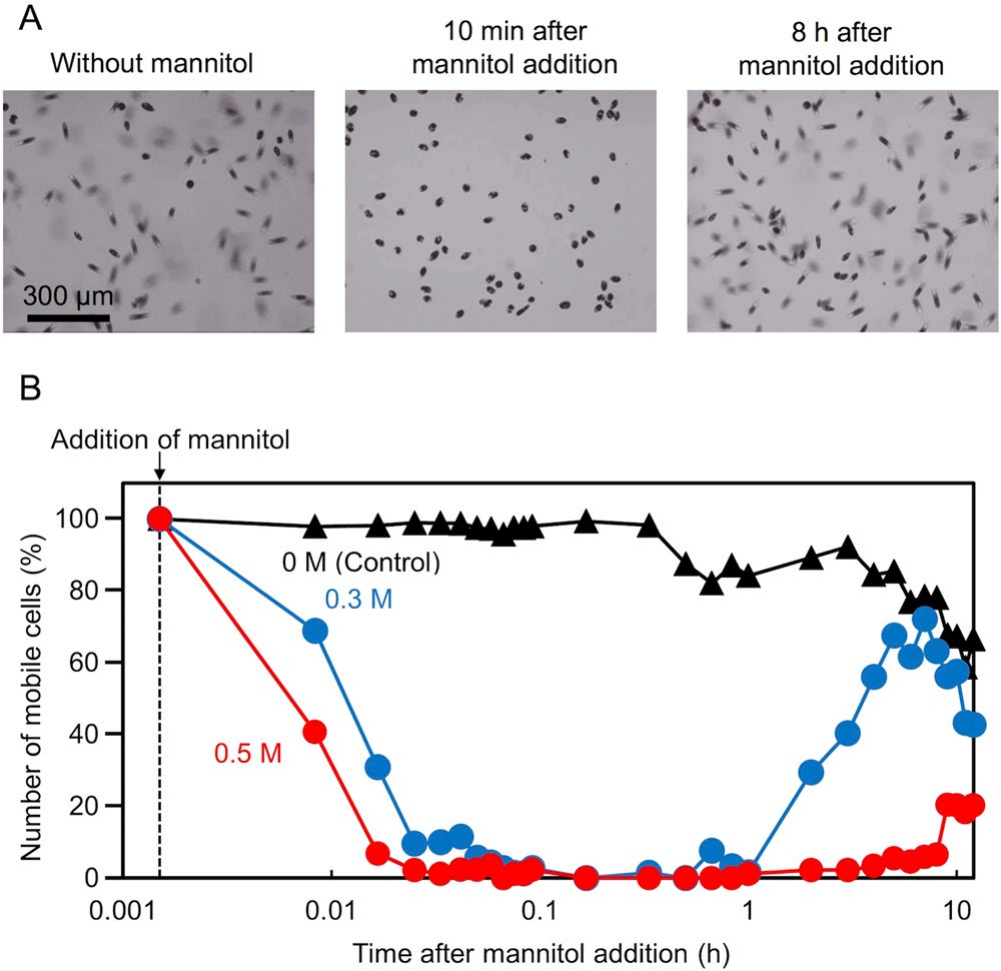
Suppression of the mobility of *E. gracilis* cells by mannitol addition. (**A**) Mobility suppression. Without mannitol, the cells freely moved in 3D and went out of the focal plane of the microscope, due to which some of the cells look out of focus and blurry. 10 min after adding mannitol to the culture medium, all the cells sank to the bottom of the petri dish and were seen in focus under the microscope. 8 hours after the mannitol addition, the cells restored their mobility and swam out of focus. (**B**) Time-varying population of control cells and mobile cells with respect to the entire cell population after the mannitol addition at different mannitol concentrations. The seemingly decreased population of control cells after 0.3 h is due to the fact that some active cells moved out of the field of view.

To optimize the photoporation, we suppressed the mobility of *E. gracilis* cells by adding mannitol to the culture medium. Under a normal culture condition without mannitol, cells freely move with their flagellates in the medium and go out of the microscope’s focal plane (Fig. 3A). After we added mannitol with a concentration of higher than 0.25 M, cells stopped their migration and sank to the bottom of the petri dish within 10 min (Fig. 3A). At a mannitol concentration of higher than 0.5 M, the mobility of all the cells was completely suppressed. This is presumably due to the hyperosmotic pressure induced by the submolar concentration of mannitol which is known to inhibit the ciliary beat of respiratory epithelium as a result of deactivating protein kinase A^35^. The number of mobile cells as a function of the concentration of the added mannitol is shown in Fig. 3B, which indicates that the mobility suppression is increased as the mannitol concentration is increased. As shown in Fig. 3A and B, about 75% of the cells (nearly 100% compared with the control) restored their mobility several hours after adding mannitol at a concentration of 0.3 M. The figure indicates that the optimum concentration is 0.3 M for the sake of cell viability.

### Femtosecond laser photoporation of *Euglena gracilis* cells at single-cell resolution

**Figure 4:**
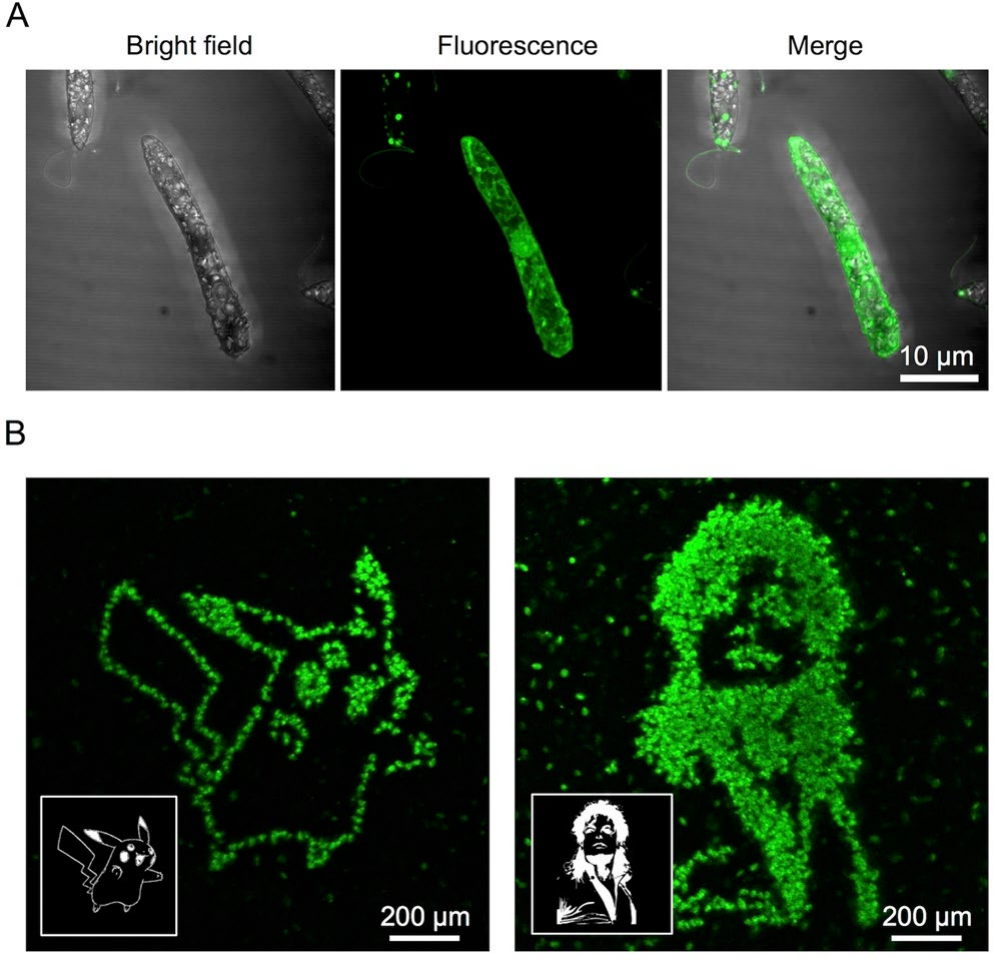
Femtosecond laser photoporation of E. gracilis cells at single-cell resolution. (**A**) Bright-field and fluorescence images of an *E. gracilis* cell 20 min after the photoporation with the FPBP. (**B**) Fluorescence images of *E. gracilis* cells 20 min after the spatially patterned photoporation with the same aptamer. The patterned photoporation was performed on the cells in the black and white patterns of Pikachu (left) and Michael Jackson (right) as shown in the insets. Each fluorescent dot corresponds to a single *E. gracilis* cell into which the aptamer was injected and bound to intracellular paramylon. The images firmly show the demonstration of the photoporation with the single-cell resolution.

For the femtosecond laser photoporation, we focused femtosecond laser pulses onto cells to transiently perforate the cell membrane of *E. gracilis*. Here we used chloroplast-free *E. gracilis* cells to avoid a spectral overlap between autofluorescence from intracellular chloroplasts and fluorescence from NBD in the aptamer. The pulse energy and repetition rate of the laser were 80 nJ and 1 kHz, respectively while the diameter of the laser focus on the cells was about 2 µm. We scanned the laser spot on the cells on a petri dish by controlling a motorized stage on a microscope at a speed of 100 µm/s. Figure 4A shows a fluorescence image of a photoporated *E. gracilis* cell observed under a laser-scanning confocal microscope with a 100× objective lens (NA. 1.25) where the cell was excited by continuous-wave laser light at 488 nm and imaged through a 510–540 nm band-pass filter after the laser excitation. The fluorescence pattern in the cell is similar to the distribution of paramylon reported previously^20^. To evaluate the paramylon-binding specificity of the aptamer, we performed the same experiment on *Chlamydomonas reinhardtii* cells which accumulate starch, but not paramylon, and verified detection of no fluorescence from the cells (Supplementary Fig. 3). Mannitol-bathed and photoporated *E. gracilis* cells were observed to be viable as shown in Supplementary Fig. 4 which indicates their mobility restoration a few hours after the photoporation.

To verify the efficient delivery and binding of the FPBP to paramylon at single-cell resolution, we performed spatially resolved fluorescence microscopy of the single cells with patterned femtosecond laser excitation. Specifically, we scanned the laser spots over a confluent monolayer of *E. gracilis* cells on the petri dish in a two-dimensional raster scanning pattern with a controlled motorized stage and mechanical optical shutter. Figure 4B shows fluorescence images of many photoporated cells 20 min after the patterned excitation of black and white images of Pikachu and Michael Jackson in the insets. The images firmly show the photoporation at single-cell resolution.

### Monitoring the time-varying distribution of intracellular paramylon in *Euglena gracilis* cells

**Figure 5:**
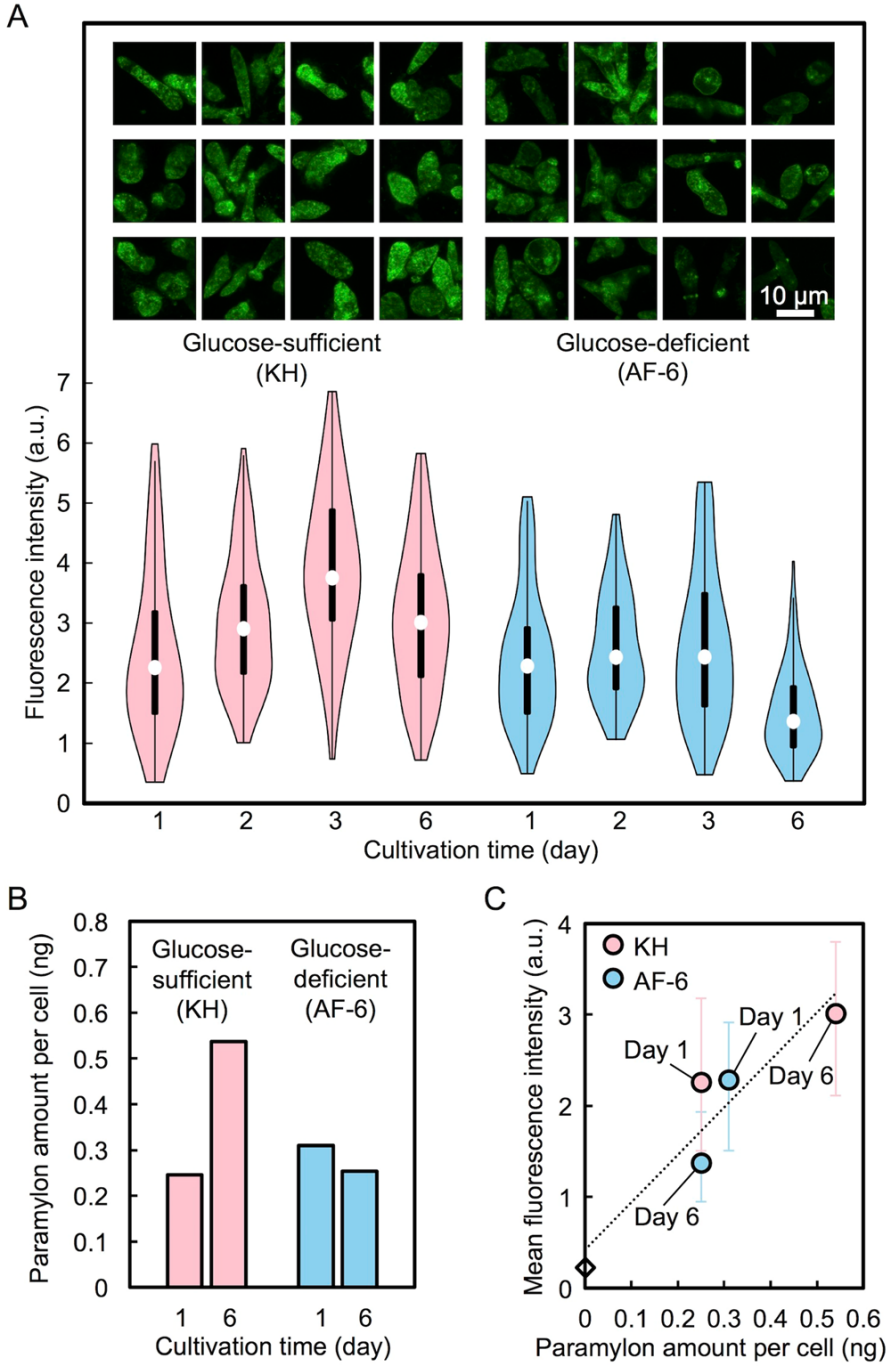
Monitoring the time-varying distribution of intracellular paramylon in *E. gracilis* cells under glucose-sufficient and -deficient conditions. (**A**) Fluorescence images (top) and violin plots of the fluorescence intensity (bottom) of *E. gracilis* cells on Day 1, Day 2, Day 3, and Day 6. (**B**) Paramylon amount per cell on Day 1 and Day 6 measured by carbohydrate assay. (**C**) Relation between the paramylon amount per cell and the mean fluorescence intensity of *E. gracilis* cells. The mean fluorescence intensity at a paramylon amount of 0 ng indicates the background fluorescence intensity of the AF-6 medium without cells.

To characterize single live photoporated *E. gracilis* cells, we performed spatially and temporally resolved fluorescence microscopy of intracellular paramylon. Specifically, we visualized and quantitatively monitored the time-varying distribution of intracellular paramylon in single live photoporated *E. gracilis* cells cultured under glucose-sufficient and -deficient culture conditions (KH and AF-6 media, respectively) by fluorescence microscopy with the FPBP. Here we also used chloroplast-free *E. gracilis* cells to avoid a spectral overlap between autofluorescence from intracellular chloroplasts and fluorescence from NBD in the aptamer. Glucose is a sole source of carbon for the cells such that the lack of glucose suppresses the intracellular production of paramylon. Figure 5A shows fluorescence images of live photoporated *E. gracilis* cells under glucose-sufficient and -deficient culture conditions on Day 1, Day 2, Day 3, and Day 6. Overall, a gradual increase in the amount of intracellular paramylon and its localized accumulation until Day 3 are evident only under the glucose-sufficient condition whereas the paramylon amount in the cells under the glucose-deficient condition was relatively intact. This overall response of the *E. gracilis* cells can be more pronounced by statistical analysis of the fluorescence images of the cells as shown in the violin plots in Fig. 5A. The reason why the fluorescence intensity of the cells in the glucose-sufficient culture was decreased on Day 6 is the depletion of glucose in the medium. In order to verify the actual increase of intracellular paramylon, we calibrated the fluorescence intensity of the cells by quantifying the amount of intracellular paramylon by carbohydrate assay, the phenol-sulfuric acid method. Figure 5B shows the averaged paramylon amount per cell given by diving the total amount of paramylon extracted from the cells by the number of the cells. This is further supported by the linear relation between the paramylon amount per cell and the mean fluorescence intensity of the cells as shown in Fig. 5C. Since every photoporated cell emitted fluorescence from the whole cell body, we think that the FPBP was sufficiently delivered into the cell so that its binding with paramylon was saturated. This is reflected by the statistical analysis in Fig. 5A that indicates the cell-to-cell differences in the intracellular paramylon amount regardless of the uncertainty in the delivery efficiency of the FPBP.

## Discussion

These results firmly show that our method enabled the specific visualization of a target intracellular metabolite, such as paramylon in our case, using fluorescence microscopy and the spatially and temporally resolved quantitative analysis of single living cells as one of its practical utilities. This ability is powerful as it is expected to reveal the anatomical behavior of the metabolite production and degradation in each living cell of Euglenids. Furthermore, it can display cellular heterogeneity in the metabolite’s intracellular accumulation and be useful for selecting high-performance cells. Because the quantity of the metabolite is measured as the intensity of fluorescence, our staining method is also applicable to flow cytometry and fluorescence-activated cell sorting. Finally, the remarkable advance of high-speed imaging and sorting technologies may assist the efficient selection of single cells with high specificity. With all these advantages and capabilities, our method is essentially superior to other population-averaged ensemble measurement methods such as chromatography, mass spectrometry, and microplate readers and holds great promise for highly efficient microalgae-based metabolic engineering.

## Methods

### Preparation of chloroplast-free *Euglena gracilis* cells

The chloroplast-free *E. gracilis* strain SM-ZK, a streptomycin-bleached mutant derived from strain Z^36^, was stocked in Koren-Hunter (KH) medium (pH 3.5)^37^ under 14/10 light/dark cycle illumination (130–150 μmol m^−2^ s^−1^) at 26 °C. Chloroplast-free *E. gracilis* cells used for the experiments were prepared by replacing the KH medium with fresh KH medium (Fig. 5A–C), AF-6 medium (Fig. 5A–C)^38,39^, or AF-6-N+ glucose medium (nitrogen-omitted AF-6 containing 12 g/L of glucose) (Fig. 4A,B) and culturing the cells for 6 days in dark.

### Library preparation for the FPBP selection

We selected the FPBP using the selection method reported in a previous report^29^ with some modifications. For the selection, we prepared the following two single-stranded DNA libraries which encode the amber stop-codon (TAG) at the center of each library: 5′-TGCGTCCGTCTCGTATGACAACATGT(VVN)_5_TAG(VVN)_5_TGTTCTTGGGACAAGAGACGGTCAGC-3′ and 5′-TGCGTCCGTCTCGTATGACAACATGT(NNK)_5_TAG(NNK)_5_TGTTCTTGGGACAAGAGACGGTCAGC-3′, where V is A, G, or C, N is A, T, G, or C, K is G or T, and the underlines represent the restriction site of BsmBI. These templates and all primers used below were purchased from Eurofins Genomics. The ssDNA libraries were annealed and double-stranded by single-cycle PCR using a reverse primer (Rev_Lib_BsmBI_150528, 5′-GCTGACCGTCTCTTGTC-3′) and PrimeSTAR GXL DNA polymerase (Takara Bio). The PCR products were purified using NucleoSpin® Gel and PCR Clean-up kit (Takara Bio) and subsequently digested with BsmBI (New England Biolab). We ligated these libraries with the following two dsDNAs using T4 DNA Ligase (Mighty Mix, Takara Bio): the first dsDNAs containing the T7 promoter and Shine-Dalgarno sequence which were ligated to the 5′ terminal of the library and the second dsDNAs containing a helical linker, TolA, and ribosome arrest sequence, SecM, which were ligated to the 3′ terminal of the library (Supplementary Fig. 5). The ligated products were amplified by PCR using the forward and reverse primers (Fwd_pCR-T7_150731, 5′-CGAAATTAATACGACTCACTATAGGGAGACCACAACGGTTTC-3′ and Rev_TolA_110617, 5′-TTAGCTCACCGAAAATATCATCTG-3′). We gel-purified the PCR products and used them as templates for the aptamer selection below.

### Aptamer selection

The mixture of the two templates at a molar ratio of 1:1 was transcribed and subsequently subjected to DNase treatment using the MEGAscript T7 transcription kit (Life Technologies). After purification using an mRNA clean and concentrator kit (Zymo Research), we mixed the mRNA with a reconstituted *in vitro* translation system (25 µL)^30^ in which RF1 was absent. We also added 14% w/w of an RNase inhibitor (RNasin Plus, Life Technologies) and 400 pmole of UAG-suppressor tRNAs carrying NBD-amPhe in the translation system. The tRNA was synthesized as previously described^29^. After 15 min of incubation at 37 °C for translation, we added 200 μl of the ice-cold WBT-RNasein buffer (WBT; 50 mM Tris/acetate, 150 mM NaCl, 50 mM magnesium acetate and 0.05% Tween 20, 5–7% RNasein Plus, pH 7) and 1 mg of paramylon granules, extracted and purified from *E. gracilis* as previously reported^40^, which were pre-washed 3 times with the WBT-RNasin buffer. The mixture was gently shaken for 30 min at 4 °C. To remove any unbound complexes, we washed the paramylon granules 8 times with the ice-cold WBT-RNasin buffer. We eluted mRNA bound to the paramylon granules using the elution buffer (50 mM Tris-acetate buffer pH7.5, 150 mM NaCl, 50 mM EDTA). After purification of the mRNA using the mRNA clean and concentrator kit, the mRNA was reverse-transcribed to cDNA (PrimeScript™ Reverse Transcriptase, Takara Bio). We amplified the cDNA using PrimeSTAR GXL DNA polymerase and primers (Fwd_pCR-T7 150731 and RevtolA110617). After purification using NucleoSpin® Gel and PCR Clean-up kit, we used the PCR product as the template for the next round of selection. We started negative selection at the fifth round of selection such that we added 20 mM of glucose in the affinity selection and washing process to eliminate glucose binders. After the seventh round of selection, we sequenced 96 clones and obtained 51 sequences (Supplementary Table 1). The five sequences were synthesized by solid-phase chemistry at the RIKEN Brain Science Institute.

### Fluorogenic properties of the peptide aptamers

We examined the fluorogenic properties of the five peptides against paramylon granules using fluorescence imaging. The five synthesized peptides were dissolved in dimethyl sulfoxide (DMSO). The concentration of each peptide was determined using the extinction coefficient of NBD, 25,000 M^−1^ cm^−1^, at an optical wavelength of 475 nm^41^. We prepared the mixtures of the peptides and paramylon granules: 5 μM of peptides (5% DMSO) and 10 mg/mL of WBT-washed paramylon granules. After 30 min of incubation at 25 °C, we observed their blight-field and fluorescence images under an inverted microscope (IX71, Olympus) equipped with a 10× objective lens (CPlan, NA. 0.25, Olympus). We used two band-pass filters, 470–490 nm for excitation and 510–550 nm for emission, to detect NBD’s fluorescence.

### Binding affinity of the FPBP

We determined the apparent binding affinity of the peptide aptamer against water-insoluble saccharides including paramylon and Kuzuko. We prepared solutions containing 10 mg/mL of pre-washed paramylon and Kuzuko and the aptamer at different concentrations of 0–50 μM in the WBT buffer. After 15 min of gentle shaking, we set the solutions in a plate reader (EnSpire 2300, Perkin Elmer). After shaking the plate for 20 s at 300 rpm in double-orbital motion, we measured the intensity of the fluorescence from the solutions (λ_ex_ = 475 nm, λ_em_ = 535 nm). We normalized the fluorescence intensity with the intensity of 100 µM of a Ru(bpy)_3_Cl_2_ solution which was also placed in the same plate.

### Femtosecond laser photoporation

The femtosecond laser photoporation was performed using the system shown in Supplementary Fig. 6. Femtosecond laser pulses from a regeneratively amplified Ti:Sapphire femtosecond laser (800 ± 5 nm, 100 fs, <1 mJ/pulse, 1 kHz) (Solstice Ace, Spectra-Physics) were used for the photoporation under an inverted microscope (IX71, Olympus). The irradiation was controlled by a mechanical optical shutter (Σ-65GR, Sigma Koki). The laser pulse energy was tuned by a neutral-density filter. The focal point of the laser was tuned onto the plane of the image on the microscope via dual convex lenses. The laser beam was focused on the cell through a 20× objective lens (UMPlanFl, NA. 0.45, Olympus) and usually scanned at a speed of 100 µm/s by a motorized microscope stage (BIOS-102T, Sigma Koki) equipped on the microscope. The optimized pulse energy was 80 nJ, which was a little higher than the threshold energy of laser-induced breakdown in the medium (70 nJ). The motorized stage and mechanical shutter were connected with a computer to produce a photoporation pattern on the cells. The interval between the consecutive laser spots on the cells was 100 nm, which is given by dividing the laser scanning speed (100 µm/s) by the pulse repetition rate of the laser (1 kHz). For the excitation pattern (Fig. 4B), two-dimensional raster scanning was performed on the confluent monolayer of the cells on the petri dish by controlling the motorized stage and mechanical shutter. The scanning speed in the horizontal (X) direction was 100 µm/s while the interval between the horizontally scanned lines in the vertical (Y) direction is 0.5 μm.

### Analysis by fluorescence imaging

After the photoporation, the fluorescence pattern of NBD in the peptide aptamer upon binding to intracellular paramylon was imaged by a laser-scanning confocal microscope (Supplementary Fig. 6). Fluorescence imaging was sequentially performed on the microscope. A 488-nm diode-pumped solid-state (DPSS) laser (40 mW) (PC14763, Spectra-Physics) was used as an imaging light source and scanned over the sample by a confocal laser scanning unit (FV300, Olympus). The laser power was tuned to 0.5 mW by a neutral density filter after the objective lens. The green portion of the fluorescence signal was detected through a 510–540 nm band-pass filter. Objective lenses of 100× (Plan, NA. 1.25, Oil, Olympus) and 10× (UMPlanFl, NA. 0.25, Olympus) were used for narrow-field, high-resolution imaging (Fig. 4A) and wide-field, low-resolution imaging (Fig. 4B), respectively. A 100× objective lens (LUMPlanFl, NA. 1.0, Water, Olympus) was used for high-resolution imaging (Fig. 5A). The fluorescence images were analyzed by the open-source image analysis tool, ImageJ ver. 1.48 v^42^. In the image analysis, the cell area was extracted from the image while the integrated fluorescence intensity in the area was evaluated. The average of the fluorescence intensity was estimated as an index of the concentration of intracellular paramylon by dividing the integrated fluorescence intensity by the cell area. The violin plot in Fig. 5A was constructed by data analysis software, R ver. 3.3.3^43^.

### Quantification of intracellular paramylon

*E. gracilis* cells were dried in a freeze dryer (Lyph-Lock 6, Labconco) after counting the cells. Paramylon in the dried cells was extracted and quantified by using the phenol-sulfuric acid method^44,45^. The average amount of intracellular paramylon per cell was estimated by dividing the total extracted amount by the cell population.

### Data availability

The data generated in this study is available from the corresponding authors upon request.

## Electronic supplementary material

Supplementary figures and table
