# Supplementary material for: Targeted delivery of fluorogenic peptide aptamers into live microalgae by femtosecond laser photoporation at single-cell resolution
Source: Sci Rep. 2018 May 29;8:8271. doi: 10.1038/s41598-018-26565-4 (PMC5974127; doi:10.1038/s41598-018-26565-4)
Supplement: Supplementary file 1 — Supplementary figures and table [file 41598_2018_26565_MOESM1_ESM.docx]

**Supplementary Information for**

**Targeted delivery of fluorogenic peptide aptamers into live microalgae by femtosecond laser photoporation at single-cell resolution**

Takanori Maeno^a^, Takanori Uzawa^b,c,1^, Izumi Kono^c^, Kazunori Okano^a^, Takanori Iino^a^, Keisuke Fukita^a^, Yuki Oshikawa^a^, Taro Ogawa^d^, Osamu Iwata^d^, Takuro Ito^e^, Kengo Suzuki^d^, Keisuke Goda^e,f,2^, and Yoichiroh Hosokawa^a,3^

*^a^ Graduate School of Materials Science, Nara Institute of Science and Technology, Ikoma 630-0192, Japan*

*^b^ Nano Medical Engineering Laboratory, RIKEN, Wako 351-0198, Japan*

*^c^ RIKEN Center for Emergent Matter Science, Wako 351-1098 Japan*

*^d^ euglena Co., Ltd, Yokohama 230-0046, Japan*

*^e^ Japan Science and Technology Agency, Kawaguchi 332-0012, Japan*

*^f^ Department of Chemistry, University of Tokyo, Tokyo 113-0033, Japan*

*^1^ E-mail: tuzawa@riken.jp; phone: +81-48-467-9302, ^2^ E-mail: goda@chem.s.u-tokyo.ac.jp; phone: +81-3-5841-4329, ^3^ E-mail: hosokawa@hskw.jp; phone: +81-743-72-6199*


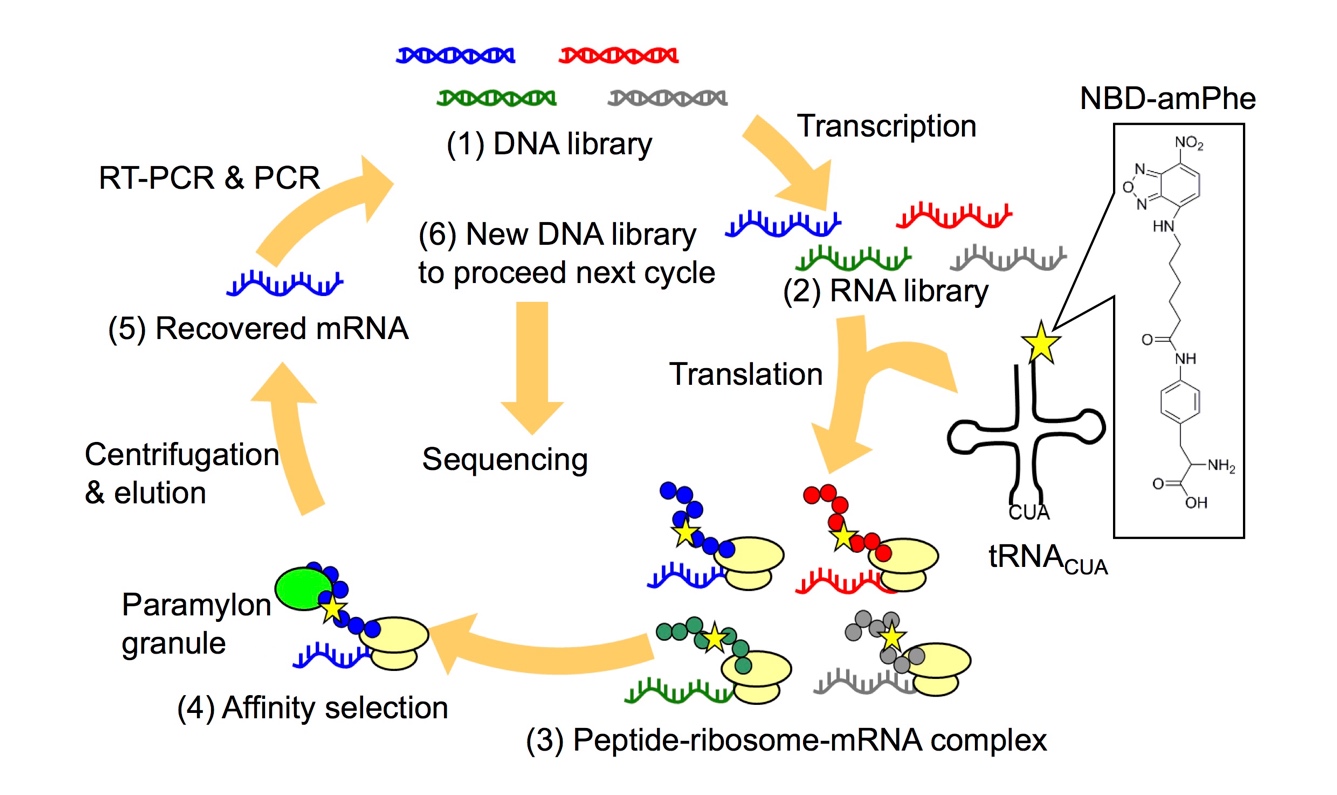


**Supplementary Figure 1 | Selection of a fluorogenic peptide aptamer using ribosome display.** We selected a fluorogenic peptide aptamer using ribosome display with modification to incorporate an NBD-coupled aminophenylalanine (NBD-amPhe). At the end of seven rounds of selection, we cloned the enriched library to read their sequences.


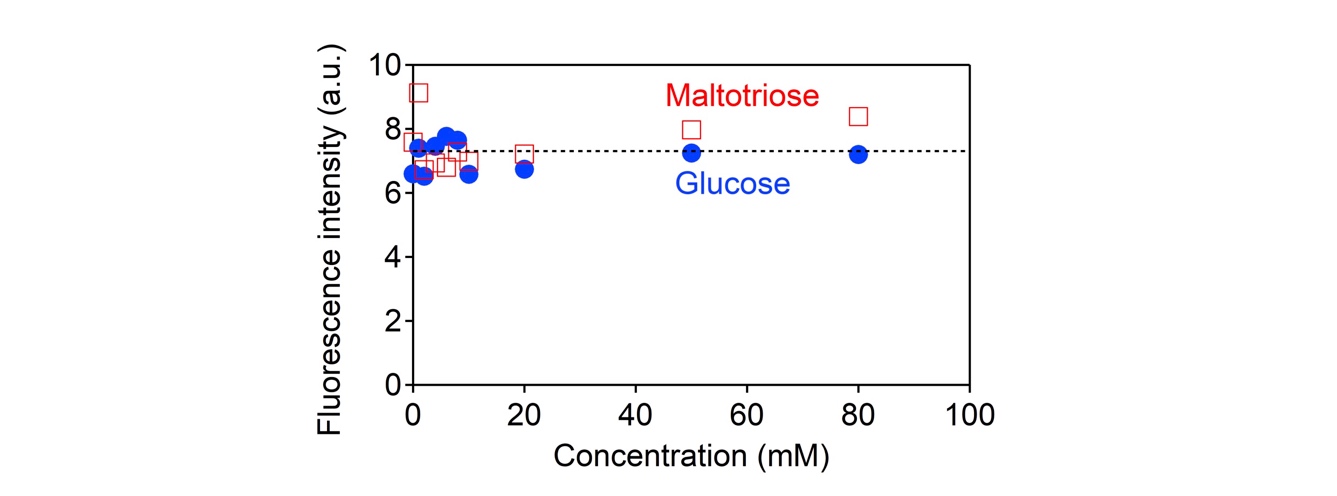


**Supplementary Figure 2 | Specificity of the FPBP.** The fluorescence signal level of the FPBP was not increased when changing the concentration of glucose nor tri-saccharide which consists of three glucoses linked with α-1,4 glycosidic bonds (maltotriose). We prepared 5 μM of FPBP and sugars at various concentrations (from 0 to 80 mM) in WBT buffer. We measured the fluorescence intensity at 543 nm by blue-light excitation at 470 ± 10 nm using a NanoDrop 3300 fluorospectrometer (Thermo Scientific, Hercules).


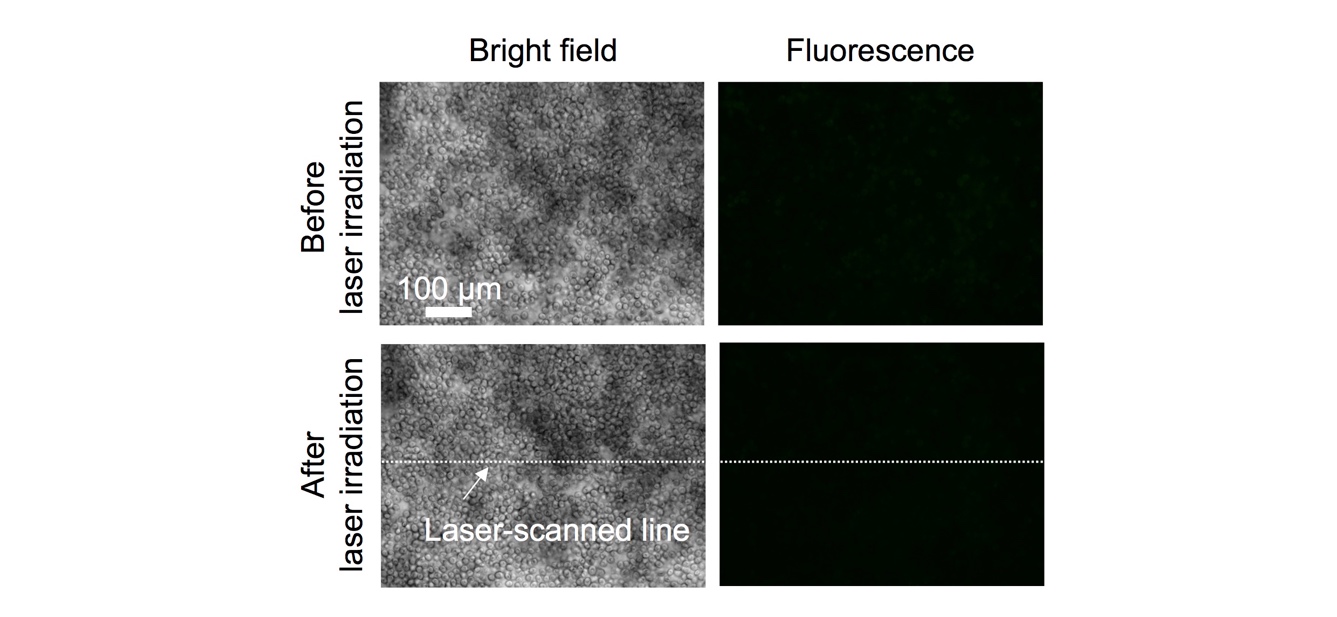


**Supplementary Figure 3 | Femtosecond laser photoporation of *Chlamydomonas reinhardtii* cells.** The cells (1×10^6^ cells/mL suspended in AF-6 medium) were mixed with the same medium containing mannitol (0.3 M) and FPBP (50 µM). Bright-field and fluorescence images of *Chlamydomonas reinhardtii* cells before and after the linearly scanned photoporation with the FPBP and femtosecond laser (200 nJ/pulse, 1 kHz) are shown in the figure.

**
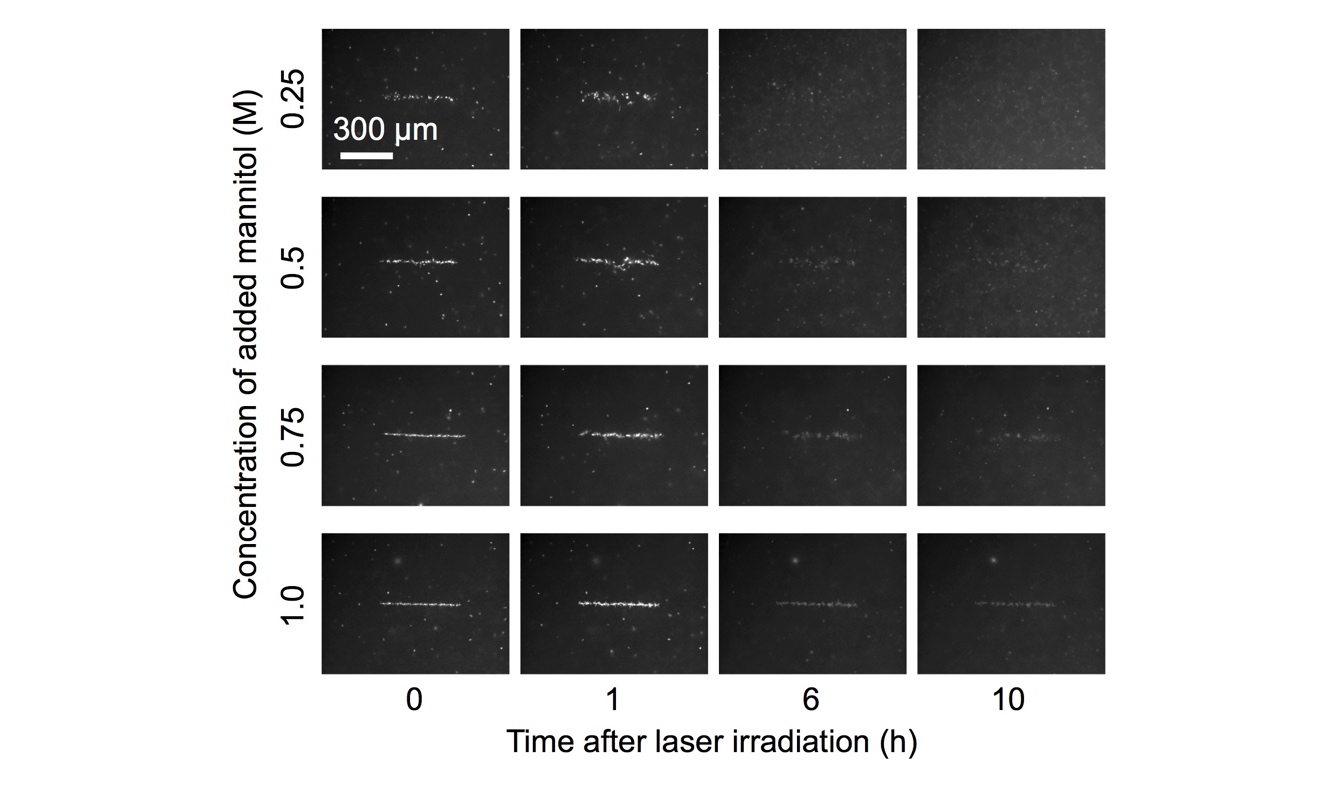
**

**Supplementary Figure 4 | Time-varying diffusion of *E. gracilis* cells after the photoporation as a function of mannitol concentration.** The fluorescence images show photoporated *E. gracilis* cells at lower concentrations of added mannitol restored their mobility while they remained immobilized for longer periods of time at higher concentrations. The cells were photoporated by the femtosecond laser in the linear scan shown in the figure. The pulse energy was tuned to be 200 nJ/pulse, which was a little higher than the threshold energy for laser-induced breakdown in the medium (180 nJ). The difference in pulse energy between this experiment and the experiments in Fig. 4a, Fig. 4b, and Fig. 5a is due to differences in experimental conditions (e.g., pulse duration, laser alignment, microscope use). In order to ensure intracellular delivery with high cell viability under such varying experimental conditions, we typically calibrate the pulse energy at the photoporation point (laser-focused point) by using the threshold energy for laser-induced breakdown. The fluorescence signal was decreased after 6 hours due to photobleaching.

**
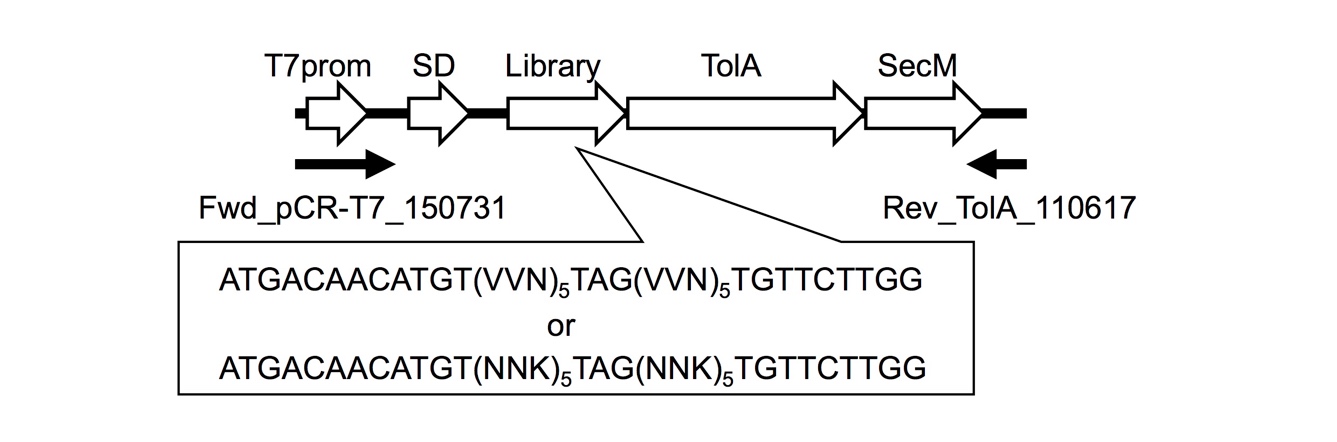
**

**Supplementary Figure 5 | Library preparation for the FPBP.** To select a fluorogenic peptide aptamer, we used the dsDNAs containing the T7 promoter, Shine-Dalgarno, random library, TolA (a helical linker), and SecM (ribosome arrest) sequences.

**
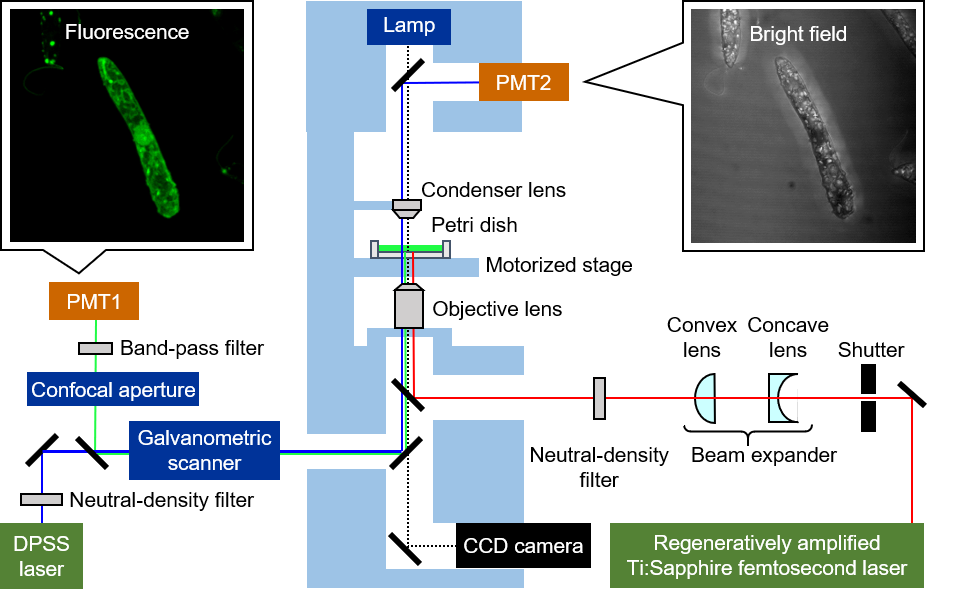
**

**Supplementary Figure 6 | Femtosecond laser photoporation system.** Femtosecond laser light (800 nm, 150 fs) was introduced into a confocal laser scanning microscope and was focused on a *E. gracilis* cell through an objective lens. The laser pulse energy was tuned from 8 to 20 nJ/pulse. Cells in the petri dish were illuminated by the lamp with white light and monitored by the CCD camera. Confocal laser scanning microscopy of the cells was performed by using a DPSS laser, a laser scanner, a band-pass filter, and a photomultiplier tube (PMT).

**Supplementary Table 1 | Peptide sequence of the aptamer selected by ribosome display.**
